# Cellstitch: 3D cellular anisotropic image segmentation via optimal transport

**DOI:** 10.1186/s12859-023-05608-2

**Published:** 2023-12-15

**Authors:** Yining Liu, Yinuo Jin, Elham Azizi, Andrew J. Blumberg

**Affiliations:** 1 Department of Computer Science, Columbia University, New York, USA; 2 Department of Biomedical Engineering, Columbia University, New York, USA; 3 Department of Mathematics, Columbia University, New York, USA; 4 Data Science Institute, Columbia University, New York, USA; 5 Irving Institute for Cancer Dynamics, New York, USA

**Keywords:** Bioimaging, Optimal transport, 3D segmentation

## Abstract

**Background:**

Spatial mapping of transcriptional states provides valuable biological insights into cellular functions and interactions in the context of the tissue. Accurate 3D cell segmentation is a critical step in the analysis of this data towards understanding diseases and normal development in situ. Current approaches designed to automate 3D segmentation include stitching masks along one dimension, training a 3D neural network architecture from scratch, and reconstructing a 3D volume from 2D segmentations on all dimensions. However, the applicability of existing methods is hampered by inaccurate segmentations along the non-stitching dimensions, the lack of high-quality diverse 3D training data, and inhomogeneity of image resolution along orthogonal directions due to acquisition constraints; as a result, they have not been widely used in practice.

**Methods:**

To address these challenges, we formulate the problem of finding cell correspondence across layers with a novel optimal transport (OT) approach. We propose CellStitch, a flexible pipeline that segments cells from 3D images without requiring large amounts of 3D training data. We further extend our method to interpolate internal slices from highly anisotropic cell images to recover isotropic cell morphology.

**Results:**

We evaluated the performance of CellStitch through eight 3D plant microscopic datasets with diverse anisotropic levels and cell shapes. CellStitch substantially outperforms the state-of-the art methods on anisotropic images, and achieves comparable segmentation quality against competing methods in isotropic setting. We benchmarked and reported 3D segmentation results of all the methods with instance-level precision, recall and average precision (AP) metrics.

**Conclusions:**

The proposed OT-based 3D segmentation pipeline outperformed the existing state-of-the-art methods on different datasets with nonzero anisotropy, providing high fidelity recovery of 3D cell morphology from microscopic images.

**Supplementary Information:**

The online version contains supplementary material available at 10.1186/s12859-023-05608-2.

## Introduction

Spatial profiling of transcriptional states enables researchers to understand how the organization of cells influences function by providing spatial context to single cells. Indeed, spatially resolved transcriptomics was crowned as the Method of the Year in 2020 due to the valuable biological insights it provides [1]. As a first step in the pipeline, segmentation defines cell boundaries from fluorescent staining signals, assisting the assignment of RNA amplicons to reconstruct a gene-cell matrix; hence it plays a crucial role in characterizing cell types, their morphology, and location in the context of their microenvironment [2]. Segmentation is also a critical step in the analysis of multiplexed imaging of proteins [3, 4].

Deep learning has been successful at 2D cell segmentation, leveraging the availability of diverse labeled 2D training data and specialized deep learning architectures such as U-Net [5]. Trained on labeled 2D images, 2D cell segmentation pipelines such as Mesmer [6], StarDist [7], and Cellpose [8] can segment cells from 2D images with minimal human supervision and achieve expert-level accuracy. On the other hand, due to the lack of large and diverse 3D training datasets, approaches that utilize 3D neural networks [9–12] do not have comparable accuracy and generalizability, particularly in complex or dense tissues comprised of heterogeneous and abnormally shaped cells such as cancer cells. Additionally, these methods often lead to the over-segmentation of cells or noisy masks, thus impacting downstream analyses. As 3D transcriptomics studies increase in scale and computational cost [13], the need for a robust, generalizable, and user-friendly 3D cellular instance segmentation pipeline has become increasingly urgent [10]. To leverage the accuracy of 2D segmentation, [8] performs 2D segmentation layer by layer along a stitching direction and declares two cell slices as coming from the same cell if their overlap exceeds a predefined threshold. However, when cells are stacked roughly on top of each other along the stitching direction, the resulting masks are inaccurate along the non-stitching directions. Alternative approaches have been proposed to segment cell slices on different projections of the images with subsequent 3D reconstruction [8, 14], but the performance of such methods often suffers because of *anisotropy* in the microscopic images, i.e., inhomogeneity of image resolution among different dimensions due to experimental constraints. For instance, the thickness of tissue resections can be determined by imaging technology, tissue availability, and ensuring preserved tissue architecture, hence leading to higher resolution in the imaging (*X* and *Y*) dimensions than along the slicing (*Z*) dimension for softer tissues.

Here we present CellStitch, a pipeline that applies optimal transport to segment and reconstruct cells from 3D images without requiring large 3D training datasets. Optimal transport (OT) studies the best way of transforming a source distribution into a target distribution [15]; it is a natural way to pose matching problems and hence has been widely studied in mathematics, economics, and statistics. Recently, optimal transport has also been applied in the computer vision and machine learning communities because of the computational efficiency of its relaxation [16].

**Fig. 1:**
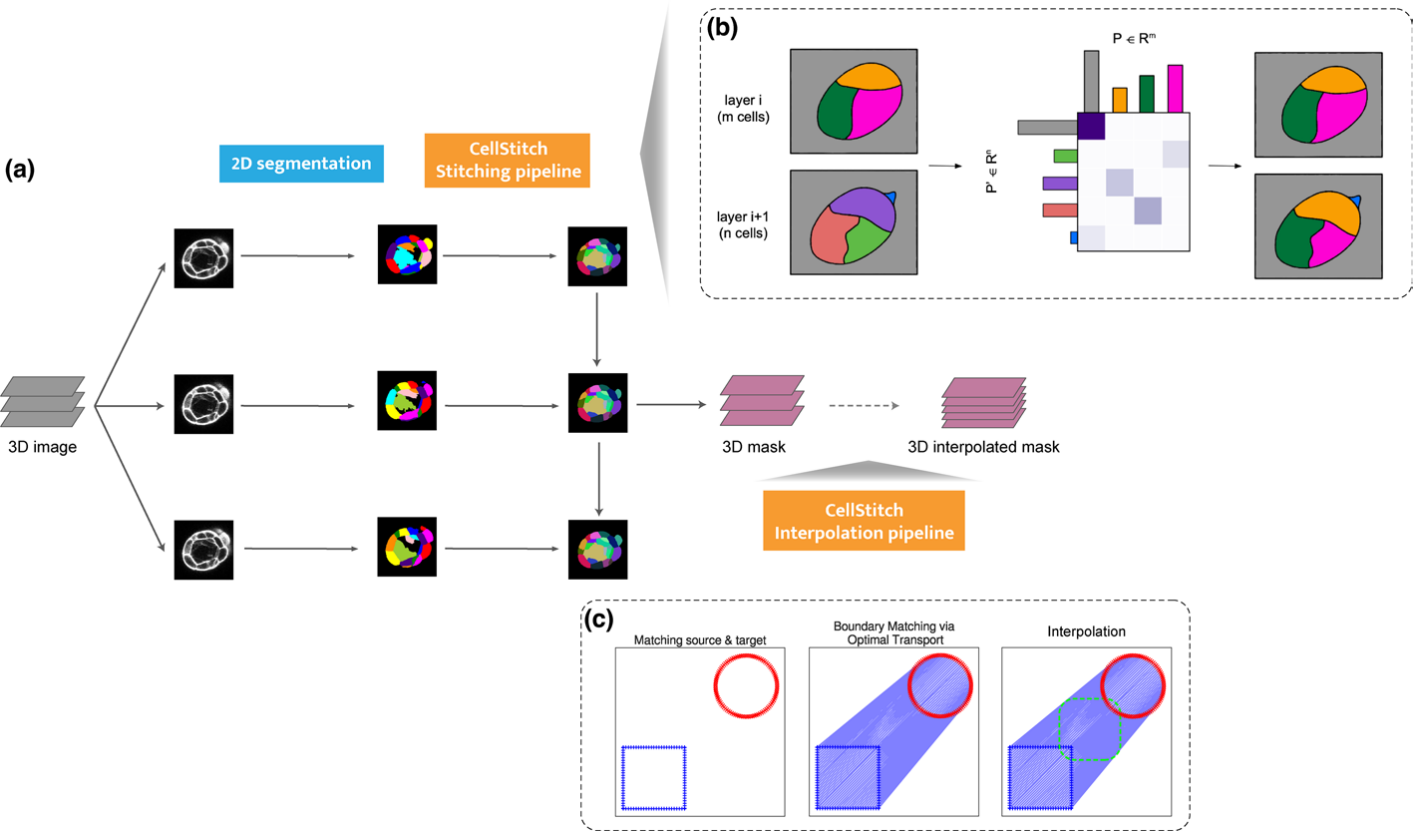
Overview of CellStitch framework. **a** CellStitch consists of a 3-step pipeline to reconstruct 3D cells: backbone 2D segmentation along *Z*-axis direction, stitching module, and an optional interpolation module. **b** Stitching pipeline: CellStitch first computes the source and target distributions based on the cell masses on given adjacent layers, and a cost matrix based on the pairwise overlap. The solved optimal transport plan is then used to deduce the optimal correspondence of cells across the adjacent layers. Finally, it reassigns instance labels of cell slices to enforce labeling consistency across all layers and creates new labels when new cells emerge based on the segmentations masks on the other two projections. **c** Interpolation pipeline: After finding optimal instance stitching, CellStitch leverages pixel-wise boundary matching followed by morphology interpolation to predict the internal layers between adjacent slices

CellStitch focuses on stitching 2D masks along one dimension obtained from any method. Throughout this paper, we leverage high-quality 2D segmentation masks obtained from Cellpose [8]. Hence, CellStitch does not require end-to-end training of any 3D network. Additionally, in contrast to a commonly-used stitching method [8] that fails to incorporate crucial information from the other directions, CellStitch uses optimal transport to trace cells across image layers and then uses segmentations from the other two directions to guide the declaration of new cells. In particular, to find correspondence between cells in adjacent layers, we model the layers as certain associated discrete distributions and obtain the optimal correspondence of cells based on the optimal transport plan. The CellStitch framework is illustrated in Fig. 1.

To summarize, the main contributions of our work are as follows:

- We formulate the problem of finding correspondence between cells from adjacent layers in terms of optimal transport using a novel cost matrix. In particular, we highlight the importance of using pairwise cell overlaps to compute the cost matrix for the given task instead of the widely-used pairwise distances.
- We design a framework to segment cells from 3D images that does not require end-to-end training of 3D networks. This is achieved by using the optimal transport plan to infer cell correspondence across different layers, and leveraging 2D segmentations from the other projections to resolve the stitching ambiguity of the matched cell slices.
- We further provide a novel interpolation framework based on optimal transport to interpolate adjacent layers; the interpolation extension can be used to reduce the anisotropy of images.
- We compare our 2D-based framework against the state-of-the-art 2.5D-based and 3D-based frameworks, and observe that CellStitch consistently performs better across datasets with anisotropy and is comparable to the best on isotropic datasets with spherical cells.

## Related work

With the recent emergence of imaging-based spatial transcriptomics and proteomics platforms [17, 18], accurate quantification of cell boundaries becomes increasingly essential. In recent years, the state-of-the-art deep convolutional neural networks (CNNs) based on U-Net [5] or ResNet backbones [19] have enabled successful 2D and 3D segmentation across various medical imaging domains [20, 21]. The increasing development of multiplexed 3D imaging technologies has driven higher demand for volumetric 3D segmentation.

To our knowledge, there are three major approaches for 3D instance segmentation: 3D end-to-end training, 2D segmentation layer by layer followed by stitching, and 2.5D projections followed by 3D reconstruction. The direct 3D approaches, such as PlantSeg [12] and 3DCellSeg [10], rely on 3D U-Net variants trained upon 3D annotated datasets. The 3D models enable smooth boundary predictions by incorporating contextual information from nearby layers in all directions. As an alternative to their high computational training costs (especially when using large language models) and given the very limited amount of annotated 3D segmentations for training, we propose a fast method to reconstruct robust 3D segmentations from segmented 2D slices.

Meanwhile, the latest 2D segmentation pipelines [6, 8] have shown robust performance aided by diverse annotated data modalities and 2D models along *XY* planes. The final 3D reconstruction is then performed by stitching the output masks along the *Z*-axis with heuristic instance mapping methods such as Intersection-over-Union (IoU). Their performance is sensitive to the empirical stitching threshold; optimal threshold values are data-dependent and hard to determine a priori. Moreover, the existing 2D-based approaches fail to resolve the stitching ambiguity imposed by the arrangement of cells. Empirically, we observe a nontrivial number of pairs of cell slices that have significant overlap but are from distinct cells, because distinct cells are stacked on top of each other; as a result, existing 2D-based approaches tend to produce under-segmented masks on the projections along the *X*- and *Y*-axes.

The 2.5D segmentation approaches [8, 14] attempt to combine the advantages of 2D and 3D, utilizing contextual layer awareness with efficient, transferable models. For instance, Cellpose3D [8] trains models to predict the flow vectors for each pixel; in order to obtain the 3D flow vectors, Cellpose3D averages the 2D flow vectors along the *XY*, *XZ* and *YZ* directions. Nevertheless, the substantial inhomogeneous sampling ratios between *XY* plane and *Z*-axis introduce noise to the segmentation pipeline, leading to over-segmentation on highly anisotropic images.

Recently, algorithms based on ideas from optimal transport theory have begun to find applications in biology [22, 23]. In particular, PASTE [24] performs pairwise spot alignment across adjacent Visium layers [25], and SCOTT [26] designs a shape-location combined system for cell tracking in 2D microscopy videos. Other applications of optimal transport have addressed 2D and 3D image registrations such as retinal fundus alignment [27, 28]. However, those methods cannot be easily applied to fluorescent images with dense, cluttered cells, because they mainly focus on spot or pixel-level alignment with single or at most a few objects of interest. Therefore, to overcome the limitations of direct 3D segmentation, CellStitch applies optimal transport to align cell objects generated from any given well-trained 2D segmentation model along the *Z* direction in order to reconstruct final 3D segmentations.

## Methods

### Background

We will give an overview of optimal transport in this section; for a more comprehensive treatment of the subject, we recommend [16]. Originally, the field of optimal transport is motivated by finding the optimal allocation of resources. A classical example is that there are factories producing products and stores demanding products. Since the sizes of the factories (and correspondingly the sizes of the stores) are different, the supply and demand is nonuniform across factories and stores, respectively. Considering the location of the factories and the stores, the cost of transporting products between each pair of factory and store is different. In this example, the goal of optimal transport is to find an allocation plan to transport products from factories to stores so that the transportation cost is minimized.

Formally, for discrete measures, the Kantorovich formulation of the optimal transport problem is as follows: given two discrete measures $P: [m] \rightarrow \mathbb {R}$ and $P': [n] \rightarrow \mathbb {R}$, a transport plan $M \in \mathbb {R}_+^{m \times n}$ is a product distribution on $[m] \times [n]$ that has *P* and $P'$ as marginals; in other words, $M_{x, y}$ describes the amount of mass in bin *x* that flows to bin *y*. Additionally, given a cost matrix $C \in \mathbb {R}^{m \times n}$ such that $C_{x, y}$ describes the price associated with moving a unit of mass from bin *x* to bin *y*, the Kantorovich optimal transport solves for

$$\begin{aligned} \hat{M}:= \arg \min _{M \in \Pi (P, P')} \langle C, M \rangle = \sum _{x, y} C_{x, y} M_{x, y} \end{aligned}$$

where

$$\begin{aligned} \Pi (P, P') = \{M \in \mathbb {R}_+^{m \times n}: \sum _{y} M_{x, y} = P, \sum _{x} M_{x, y} = P' \}. \end{aligned}$$

In order to solve the optimization problem, one can reduce it to a max-flow problem on a bipartite graph, where the vertices are cell slices in the two layers and there is an edge between two vertices if and only if the they are in different layers. The vertice and edge weights are given by the proportions of label masses and the cost matrix correspondingly. As a result, an optimal solution can be found using the max-flow algorithms.

The choice of the cost matrix *C* determines the properties of the optimal transport problem such as the uniqueness of solutions and the computational cost of solving the optimization problem. A popular choice to compute the cost matrix is the $l_p$ distance, where $l_p(x, y):= ||x - y||^p$. In this case, optimal transport induces a distance on the space of distributions, referred to as the *p*-Wasserstein distance. In particular, the 2-Wasserstein distance is obtained by using the Euclidean distance as the cost matrix; it has gained popularity in the computer vision community for performing interpolation, color transfer, and geometry processing [16, 29].

### Optimal transport for stitching adjacent layers

Suppose there are $m+1$ labels in layer *z* and $n+1$ labels in layer $z+1$ along *Z*-axis, where the label 0 is reserved for background pixels and any label $l > 0$ represents a cell label; the goal is to relabel pixels in layer $z+1$ so that the pixels corresponding to the same cell have the same label in the two layers.

In terms of optimal transport, we want to find the optimal transport plan between the two discrete distributions $P: [m+1] \rightarrow \mathbb {R}$ and $P': [n+1] \rightarrow \mathbb {R}$ where

$$\begin{aligned} P(x)&:= \frac{\hbox {number of pixels of cell} x \hbox {in layer} z}{ \hbox { total number of pixels (including background) in layer}\ z} \\ P'(y)&:= \frac{\hbox {number of pixels of cell} y \hbox {in layer} z+1}{ \hbox { total number of pixels (including background) in layer}\ z+1} \end{aligned}$$

i.e. the distributions are proportions of label masses.

The cost matrix $C \in \mathbb {R}^{(m+1) \times (n+1)}$ is defined as

$$\begin{aligned} C_{x,y} = 1 - {J(x, y), \; J(x, y) =} \frac{I(x, y)}{U(x, y)} \end{aligned}$$

where

- *J*(*x*, *y*) is the Jaccard index of *x* and *y*;
- *I*(*x*, *y*) is the number of pixels in the intersection of cell *x* in layer *z* and cell *y* in layer $z+1$;
- *U*(*x*, *y*) is the number of pixels in the union of cell *x* in layer *z* and cell *y* in layer $z+1$.

The solved transport plan $\hat{M} \in \mathbb {R}_+^{(m+1) \times (n+1)}$ tells us the optimal way of moving labels in layer *z* to labels in layer $z+1$.

**Fig. 2:**
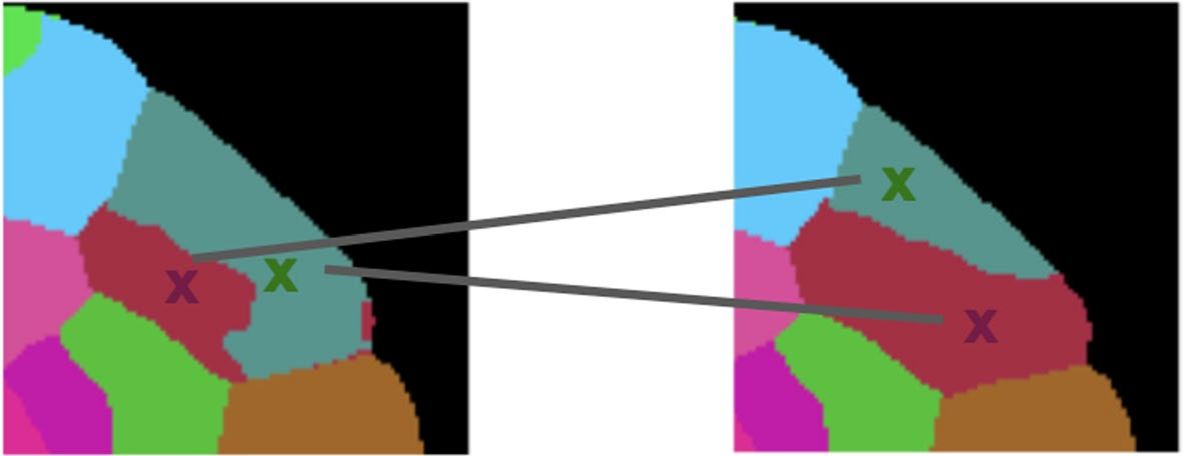
Poor correspondence of cell centroids. Using the pairwise distances between cell centroids would result in mismatched cells; as a result, this motivates the use of the union over intersection to compute the cost matrix. Here, the color code is based on the ground truth correspondence while the edges highlight the misassignments based on cell centroids. The image is taken from cell labels from the ovules dataset [31]

The Jaccard index is a similarity measure that has been commonly used as a statistic to measure the similarity of sample sets [30]; it is not, however, the most common measure used to define the cost matrix in optimal transport. The most common way of defining cost matrices are using the Euclidean distances, but in our case, using pairwise distances between cell centroids would not give us an optimal assignment. Consider the example shown in Fig. 2: the green and red cells would be incorrectly matched if we were to use the pairwise distance between the centroids as the cost matrix.

### CellStitch stitching algorithm

We now describe how we use the optimal transport plan $\hat{M}$ computed in Sect.  to relabel cells in layer $z+1$ consistently with the labels in layer *z*.

For each cell, *x* in layer *z*, notice that $\hat{y}:=\arg \max _y M_{x, y}$ is the cell in layer $z+1$ such that cell *x* most likely moves to. Define the cell tracing function $T_M: [m + 1] \rightarrow [n + 1]$ induced by the transport plan *M* as

$$\begin{aligned} T_{M}(x) = \arg \max _y M_{x, y}. \end{aligned}$$

If $T_M^{-1}(y) = \emptyset$ (i.e. no cells in layer *z* to trace the cell *y*), we declare *y* as a new cell and assign it a new label $l \not \in [n + 1]$. Note that $T_M$ might not be an injection; for a given cell in layer $z+1$, there can be more than one cell in layer *z* that traces to *y*. As a result, for each cell $y \in [n + 1]$, we match it to the cell tracing to it in layer *z* with the least cost:

$$\begin{aligned} y \mapsto \arg \min _{x \in T_M^{-1}(y)} C_{x, y}. \end{aligned}$$

As opposed to deterministic methods that match cell slices based on local IoU overlap (e.g. Cellpose2D), our formulation enables probabilistic matching learned by solving an optimal transport problem which is a global formulation that captures overlapping information of all pairs of cell slices all at once. In addition to the principled mathematical formulation, our formulation also provides a natural way of handling over-segmentation errors in layer *z*. Since we fix the labeling of layer *z* and use the labels in layer *z* to label cell slices in layer $z + 1$, when there is over-segmentation in layer *z*, there will be multiple cell slices $x_0, x_1, \cdots , x_k$ in layer *z* that trace to the same cell slice *y* in layer $z + 1$ by the tracing function $T_M$. Since we only relabel cell slice *y* in layer $z + 1$ with the one of the $x_i$’s (in particular, the one with the maximum IoU with *y*), the rest of the cell slices $x_i$’s will not be used to relabel any cell slices in layer $z + 1$. The unused cell slices $x_i$’s will then only appear in isolated *z* layers, which can be removed after a final pass through the labeled masks. On the other hand, since our procedure focuses on labeling 2D masks in a consistent manner instead of modifying the shapes of the 2D masks, the procedure will not be able to correct under-segmentation errors. We therefore advise the users to perform pre-processing steps such as increasing the contrast of the raw images as attempts to prevent under-segmentation errors in the 2D masks.

**Fig. 3:**
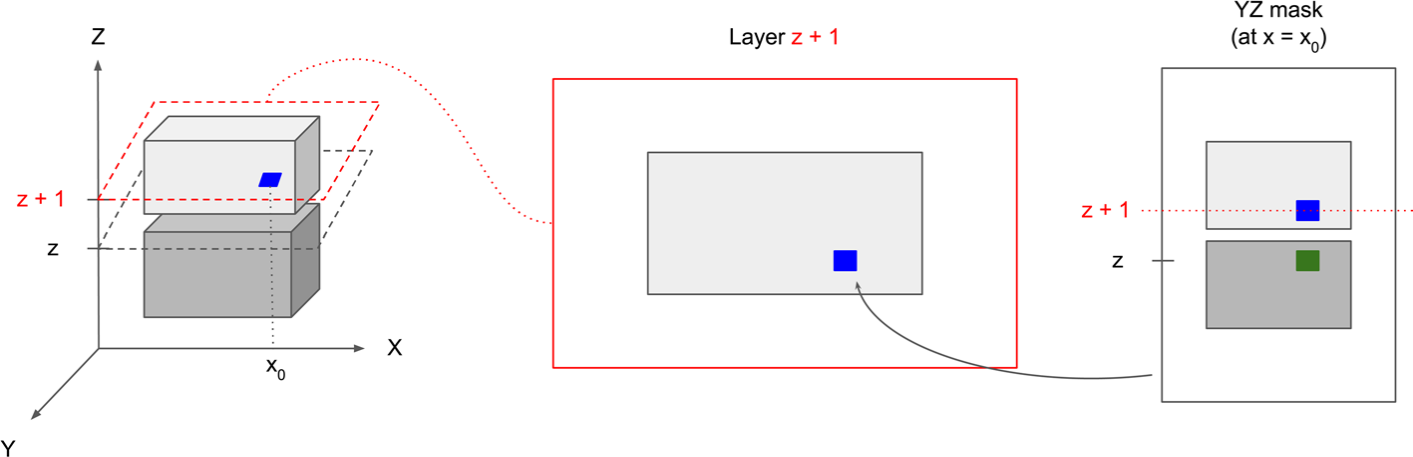
Stitching rejection voting mechanism. The diagram shows the case of how the voting mechanism prevents incorrectly stitching two cells stacked on top of each other in the *Z*-direction. Left: An example of “splitting” scenario that would have been matched by the OT formulation. The blue pixel (middle) will vote to reject stitching due to different cell labels in the corresponding index *z* & $z+1$ in the *YZ*-masks (right)

Notice that the matched cell slices should not always be assigned to the same labels, because they might come from distinct cells if the two cells are stacked on top of each other in the *Z*-axis direction. In order to resolve the ambiguity imposed by the placement of cells, we use a voting scheme to decide whether two OT-matched cell slices will be stitched or not (see Fig. 3). Each cell pixel in the matched cell in layer $z+1$ gets two votes to reject stitching. For a given pixel, we use the 2D segmentation masks (*YZ*-masks and *XZ*-masks) in the projections to *X*- and *Y*-axis to determine whether they vote for rejecting stitching or not. In particular, a cell pixel in layer $z+1$ will use one rejection vote if it gets assigned different cell label than the corresponding cell pixel in layer *z* using the *YZ*-masks (similarly for the *XZ*-masks). Intuitively, each pixel in layer $z + 1$ will use a *XZ*-mask (or *YZ*-mask) vote if the pixel does not get a same cell label as the corresponding pixel in layer z using the *XZ*-masks (i.e. projections to the *Y*-axis). In other words, we use the proportion of rejection votes as a proxy to the likelihood of two cell slices coming from two cells lying on top of each other (as opposed to coming from the same cell). At the end, the two cell slices will only be stitched (i.e. assigned as the same cell labels) if the proportion of rejection votes is smaller than a user-defined threshold. Algorithm 1 summarizes the stitching algorithm. In practice, we find that stitching from the top to the bottom layer produces nearly identical results to stitching from the bottom to the top layer; open source code implementing the stitching algorithm from the top to the bottom layer is available at https://github.com/imyiningliu/cellstitch.

**Algorithm 1:**
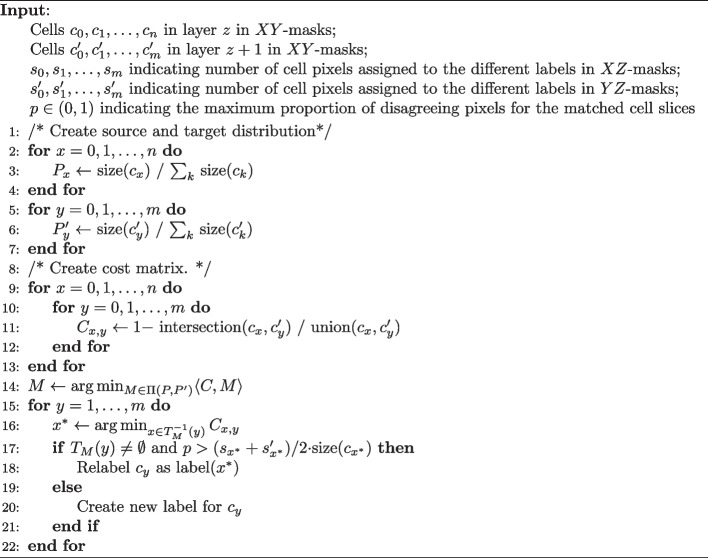
CellStitch: Stitching Algorithm

### CellStitch interpolation algorithm

Due to cost and technology constraints of 3D imaging as well as physical characteristics of tissues, the *Z*-direction resolution is oftentimes lower than that of the *XY*-plane, introducing anisotropy to the 3D cell images. However, since cells are locally cylindrical, one could hope to decrease the anisotropy of the dataset by predicting the missing layers between adjacent slices.

We now present a pixel-level OT interpolation application of our pipeline after instance-level stitching. Given matched cell $c_s$ in layer *z* and cell $c_t$ in layer $z+1$, we aim to reconstruct the shape of cells $\{c_1,\dots , c_{N-1}\}$ located in equally distributed internal layers $\{z_1', \dots , z_{N-1}' \}$ between *z* and $z+1$, assuming the anisotropy of the original images is *N* i.e. the resolution of *Z*-direction is *N* times the resolution of *X*, *Y*-directions.

**Fig. 4:**
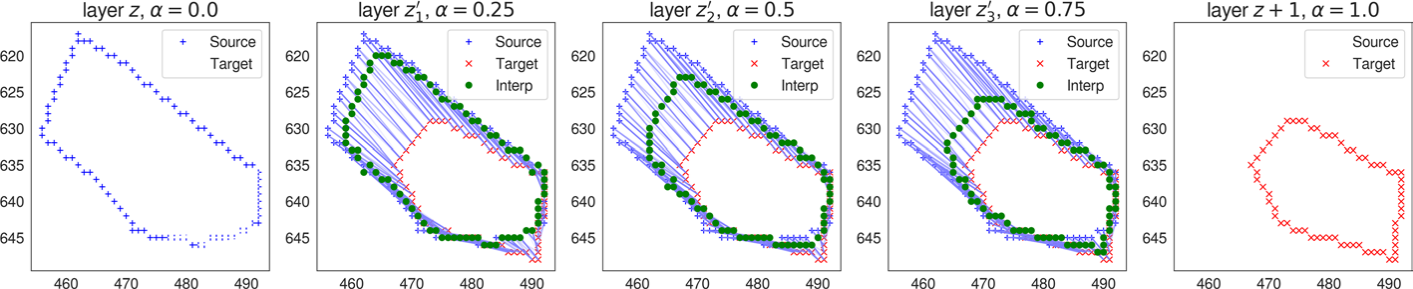
CellStitch interpolation diagram. Morphology interpolation of internal layers from an example pair of matched ovules cells in adjacent layers (anisotropy = 4)

Here we reformulate the problem as a boundary-matching task between the contour pixels across the source and target cells, and use Wasserstein interpolation [29] to infer the internal layers (Algorithm 2). In particular, in order to compute a geometry-aware average between two cell slices from adjacent layers, we first compute the optimal transport plan that achieves the 2-Wasserstein distance between the two uniform distributions on the cell boundaries. The optimal transport plan gives a partial matching between the pixels in the source cell boundary and the pixels in the target cell boundary. In order to interpolate the two slices, we compute the weighted average of the coordinates between each partially matched source and target pixel pairs, where the weights are determined by the transport plan, and further predicted the corresponding cell instances by filling the interpolated boundaries (Fig. 4). In practice, the interpolation is implemented via vectorization to avoid redundant inner-layer for loops (Algorithm 2). Open source code implementing the interpolation algorithm is also available at https://github.com/imyiningliu/cellstitch.

**Algorithm 2:**
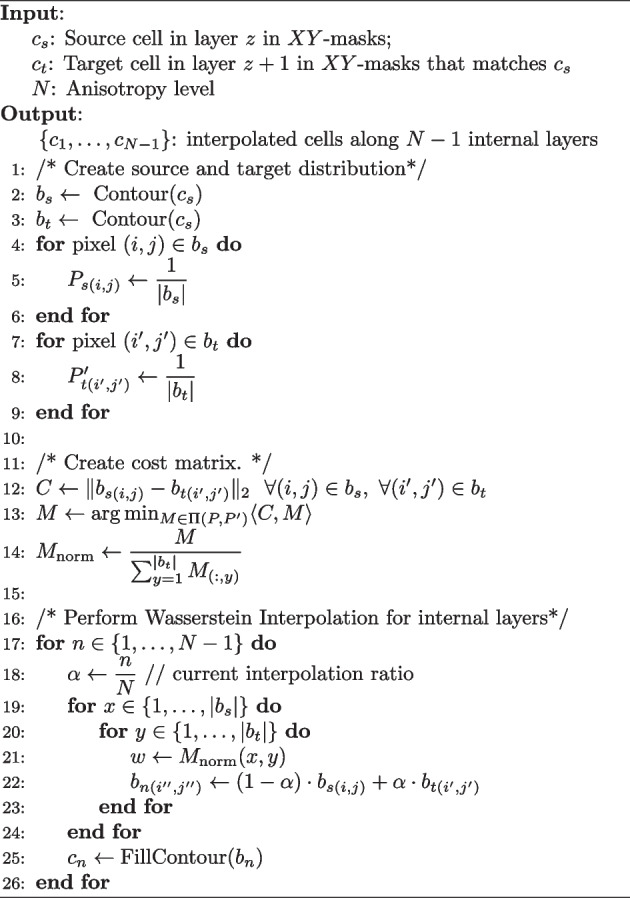
CellStitch: Interpolation Algorithm

## Results

### Datasets

We evaluated CellStitch on eight publicly available *Arabidopsis thaliana* datasets with ground-truth segmentation labels (Table 1). The first dataset (ovules) [31] contains 31 anisotropic images of ovules cells at all developmental stages using confocal laser scanning microscopy (anisotropy = 4); the ovules dataset was used to benchmark CellStitch’s stitching and pipeline performance. The second dataset (ATAS) [32] contains 125 isotropic images of apical stem cells; the ATAS dataset was used to evaluate CellStitch’s performance at increasing anisotropy levels. Finally, in order to evaluate CellStitch’s performance under a realistic setting where the anisotropy of the dataset is unknown, we further generated six additional datasets by subsampling 600 images from six different plant organs from the Arabidopsis 3D Digital Tissue Atlas (https://osf.io/fzr56).

**Table 1:** Summary of benchmarking datasets

Dataset	Number of images	Average image size ($Z \times Y \times X$ pixels)	Resolution ($Z \times Y \times X$ $\mu m$)
Ovules	31	$317 \times 910 \times 949$	$0.240 \times 0.063 \times 0.063$
Apical Stem	125	$197 \times 509 \times 509$	$0.26 \times 0.22 \times 0.22$
Anther	100	$20 \times 224 \times 224$	Unknown
Filament	100	$20 \times 224 \times 224$	Unknown
Leaf	100	$20 \times 224 \times 224$	Unknown
Pedicel	100	$20 \times 224 \times 224$	Unknown
Sepal	100	$20 \times 224 \times 224$	Unknown
Valve	100	$20 \times 224 \times 224$	Unknown

### Evaluation metrics and benchmark methods

We benchmarked our results with the state-of-the-art pipeline from each of the three classes of current deep learning-based 3D segmentation pipelines:

- Cellpose2D that stitches cell slices in adjacent layers if their IoU exceeds a user-defined threshold (2D-based) [8],
- Cellpose3D (2.5D-based) [8],
- PlantSeg’s pretrained confocal_unet_bce_dice_ds1x model (3D-based) [12].

In order to evaluate the segmentation accuracy, we first matched the cells in the segmentation mask and the ground truth label if the two cells have an intersection over union greater than *t*. Then, we computed the precision, recall, and average precision as

$$\begin{aligned} \text {precision}_{{t}}:= \frac{TP}{TP + FP}, \; \; \; \text {recall}_{{t}}:= \frac{TP}{TP + FN}, \; \; \; AP_{{t}}:= \frac{TP}{TP + FN + FP} \end{aligned}$$

where *TP* is the number of matched cells under the given threshold, *FP* is the number of unmatched cells in the segmentation mask, and *FN* is the number of unmatched cells in the ground truth labels. In the following section, we report precision, recall and AP computed at $t = 0.5$, as well as the mean average precision (mAP) averaged of $t \in \{0.25, 0.5, 0.75\}$.

We would also like to note that there are non-learning based methods that have been proposed to perform 3D segmentations; a popular non-learning segmentation method is 3D watershed [33]. In terms of dealing with anisotropy, an alternative practice is to first interpolate raw images to achieve isotropic images, and then feed the isotropic images to the segmentation pipeline. However, we have tested both 3D watershed and interpolating raw images, and found that their performance is not on par with either CellStitch or the above chosen methods. As a result, we have decided to focus on benchmarking CellStitch against the learning based methods; more details on the experimental results can be found in the supplement.

### Experimental results

We first evaluate CellStitch’s segmentation performance on the ovules dataset [31] under two settings:

- low anisotropy (anisotropy = 4): original data;
- high anisotropy (anisotropy = 8): sparsifying the *Z*-dimension by removing every other layer.

**Fig. 5:**
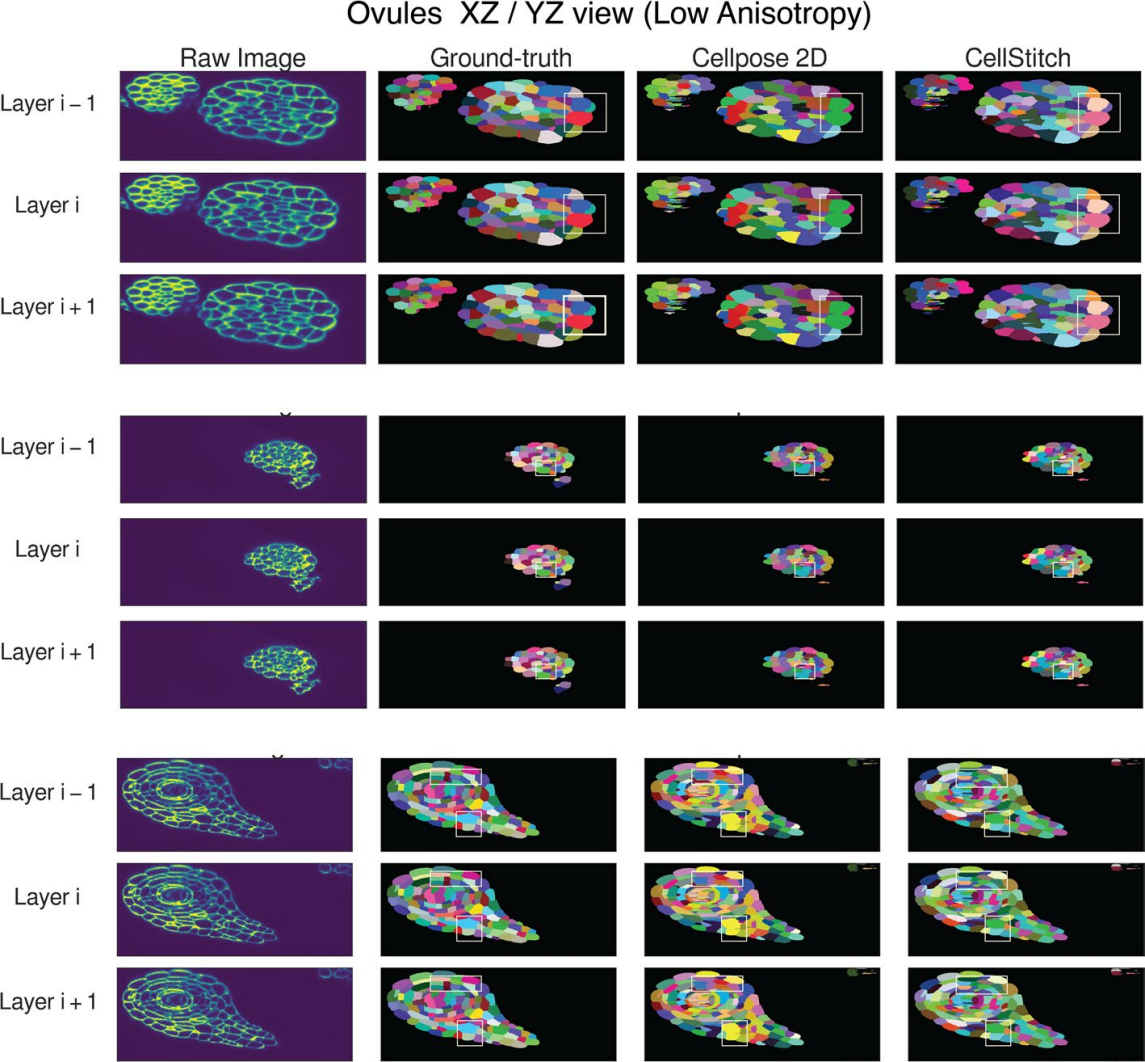
CellStitch versus Cellpose 2D. Showcases of CellStitch’s stitching rejection voting mechanism to avoid under-segmentation

**Fig. 6:**
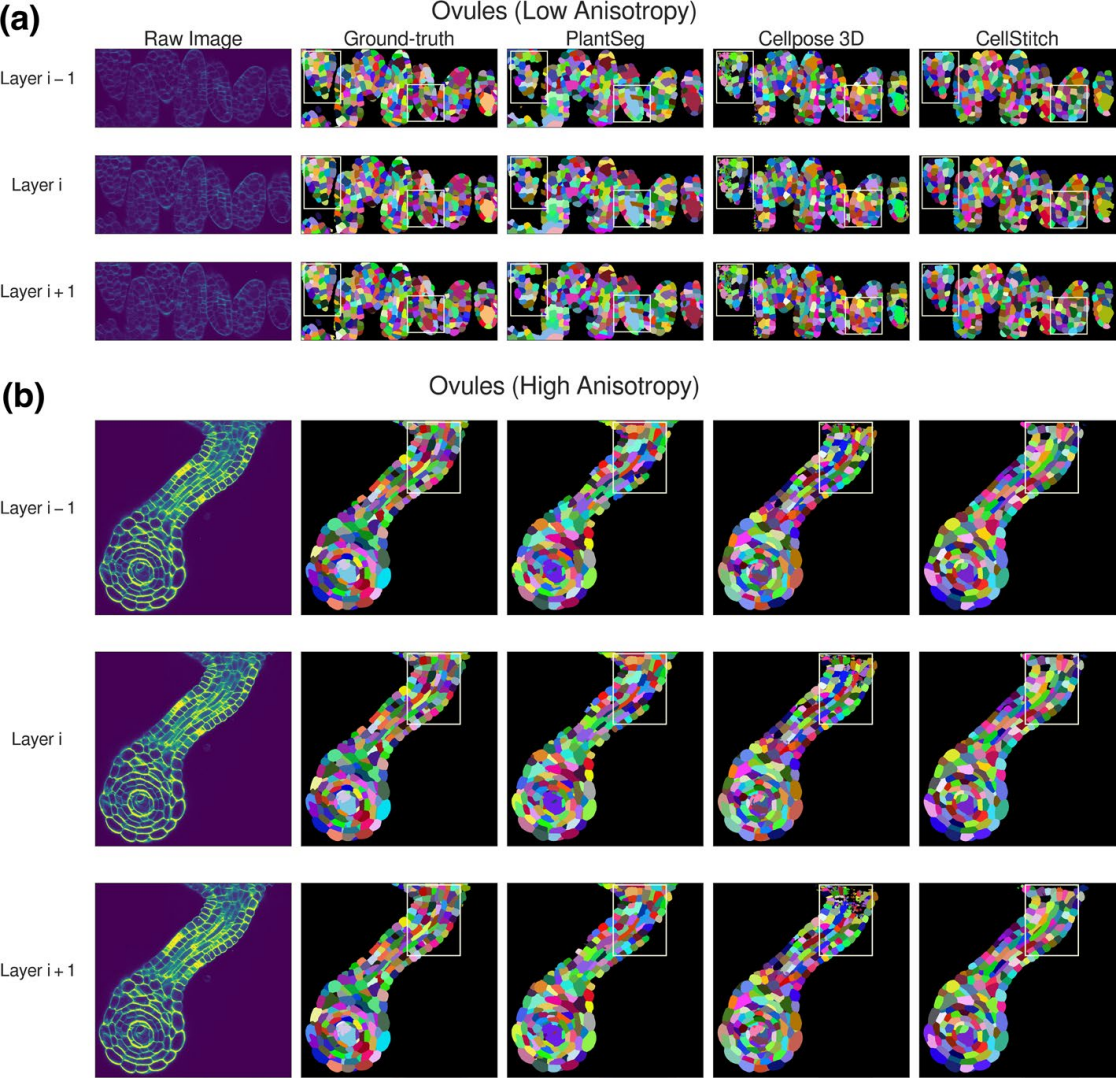
Qualitative pipeline benchmark results on consecutive Ovules slices. **a**, **b** Example low/high anisotropy confocal laser scanning microscopic images and ground-truth labels, along with PlantSeg, Cellpose3D, and CellStitch predictions are shown

To compare the performance between the 2D-based, 2.5D-based, and 3D-based methods, we used the same training data (22 training images, 2 validation images, 7 test images) that was used to train PlantSeg’s ‘confocal_unet_bce_dice_ds1x’ model to train a Cellpose 2D segmentation model for 100 epochs with learning rate 0.2 and batch size 8; the trained model was then used as a backbone to generate 2D masks later used for 3D volume reconstruction from CellStitch, Cellpose2D and Cellpose3D. We see that Cellpose3D suffers the most from increased anisotropy in the raw data, that Cellpose2D is subject to under-segmentation issues from its hard-thresholded stitching (Fig. 5), and that CellStitch consistently produces the best segmentation masks for both low and high anisotropy settings (Table 2, Fig. 6). We also benchmarked CellStitch against an example non-learning based 3D seeded Watershed, where CellStitch also demonstrates superior accuracy margin (Additional file 1: Table S1).

Then, we quantified the interpolation quality of CellStitch by reconstructing low anisotropy instances from high anisotropy images. We predicted the high anisotropy masks and further upsampled to the original *Z*-direction resolution. The interpolated masks achieved a mean average precision of 0.41; the results demonstrate that high anisotropy images achieved segmentations close to the original low anisotropy predictions of CellStitch, and both outperformed the baseline comparisons (Table 2).

**Table 2:** Performance benchmarks on the ovules dataset

Setting	Method	Precision	Recall	AP	mAP
Low Anisotropy	CellStitch$^*$	$\mathbf {0.64 \pm 0.08}$	$0.64 \pm 0.14$	$\mathbf {0.48 \pm 0.11}$	$\mathbf {0.51 \pm 0.09}$
	Cellpose2D	$0.42 \pm 0.07$	$0.57 \pm 0.09$	$0.31 \pm 0.05$	$0.36 \pm 0.05$
	Cellpose3D	$0.45 \pm 0.20$	$\mathbf {0.84 \pm 0.11}$	$0.42 \pm 0.20$	$0.42 \pm 0.20$
	PlantSeg	$0.45 \pm 0.06$	$0.80 \pm 0.07$	$0.40 \pm 0.05$	$0.41 \pm 0.05$
High Anisotropy	CellStitch$^*$	$\mathbf {0.66 \pm 0.07}$	$0.52 \pm 0.10$	$\mathbf {0.41 \pm 0.08}$	$\mathbf {0.48 \pm 0.07}$
	Cellpose2D	$0.48 \pm 0.05$	$0.54 \pm 0.09$	$0.34 \pm 0.05$	$0.40 \pm 0.04$
	Cellpose3D	$0.35 \pm 0.14$	$\mathbf {0.73 \pm 0.13}$	$0.31 \pm 0.13$	$0.31 \pm 0.12$
	PlantSeg	$0.32 \pm 0.23$	$\mathbf {0.72 \pm 0.05}$	$0.27 \pm 0.19$	$0.29 \pm 0.19$

The best performance (within 0.03) is in bold. The method that achieves the best performance under the majority of the metric is marked with $^*$

Next, we used the *Arabidopsis thaliana apical* stem cells (ATAS) dataset [32] containing 125 isotropic images under $0.22 \mu m \times 0.22 \mu m \times 0.26 \mu m$ resolution to further compare the performance of CellStitch and Cellpose; since the images in the ATAS dataset are isotropic, we are able to explore how the performance of CellStitch changes with increasing anisotropy. We followed a 7-3 train-test split to train a Cellpose model which is the backbone to generate 3D segmentations for CellStitch, Cellpose2D, and Cellpose3D; due to the lack of instructions on how to train PlantSeg models on additional datasets, we used PlantSeg’s pretrained model for the rest of the experiments. We introduced anisotropy in the ATAS dataset by subsampling across the *Z*-layers in order to further test the robustness of CellStitch and Cellpose3D against anisotropy in the dataset. We found that Cellpose3D achieves higher average precision when there is no anisotropy in the dataset, whereas Cellpose2D does not achieve comparable performance on isotropic dataset due to its ignorance of the other two directions. Additionally, similar to the results on the ovules dataset, the average precision of Cellpose3D significantly dropped under the high anisotropy setting. The quantitative results are presented in Table 3.

**Table 3:** Performance on the ATAS dataset

Setting	Method	Precision	Recall	AP	mAP
Anisotropy = 0	CellStitch	$0.79 \pm 0.08$	$0.77 \pm 0.04$	$0.64 \pm 0.07$	$0.70 \pm 0.07$
	Cellpose2D	$0.61 \pm 0.10$	$0.69 \pm 0.04$	$0.48 \pm 0.07$	$0.54 \pm 0.07$
	Cellpose3D$^*$	$\mathbf {0.87 \pm 0.17}$	$\mathbf {0.98 \pm 0.01}$	$\mathbf {0.85 \pm 0.17}$	$\mathbf {0.85 \pm 0.16}$
	PlantSeg	$0.40 \pm 0.07$	$0.92 \pm 0.16$	$0.39 \pm 0.07$	$0.39 \pm 0.07$
Anisotropy = 5	CellStitch$^*$	$\mathbf {0.81 \pm 0.04}$	$\mathbf {0.58 \pm 0.05}$	$\mathbf {0.51 \pm 0.05}$	$\mathbf {0.62 \pm 0.04}$
	Cellpose2D	$0.67 \pm 0.04$	$\mathbf {0.61 \pm 0.05}$	$0.47 \pm 0.04$	$0.56 \pm 0.03$
	Cellpose3D	$0.40 \pm 0.09$	$\mathbf {0.59 \pm 0.11}$	$0.32 \pm 0.08$	$0.32 \pm 0.06$
	PlantSeg	$0.37 \pm 0.08$	$0.31 \pm 0.13$	$0.20 \pm 0.08$	$0.21 \pm 0.06$
Anisotropy = 10	CellStitch$^*$	$\mathbf {0.75 \pm 0.04}$	$0.46 \pm 0.06$	$\mathbf {0.40 \pm 0.05}$	$\mathbf {0.53 \pm 0.05}$
	Cellpose2D$^*$	$0.64 \pm 0.04$	$\mathbf {0.55 \pm 0.05}$	$\mathbf {0.42 \pm 0.05}$	$\mathbf {0.52 \pm 0.03}$
	Cellpose3D	$0.30 \pm 0.09$	$0.38 \pm 0.11$	$0.20 \pm 0.08$	$0.26 \pm 0.06$
	PlantSeg	$0.12 \pm 0.06$	$0.04 \pm 0.03$	$0.03 \pm 0.02$	$0.00 \pm 0.00$

The best performance (within 0.03 is in bold. The method that achieves the best performance under the majority of the metric is marked with $^*$

**Fig. 7:**
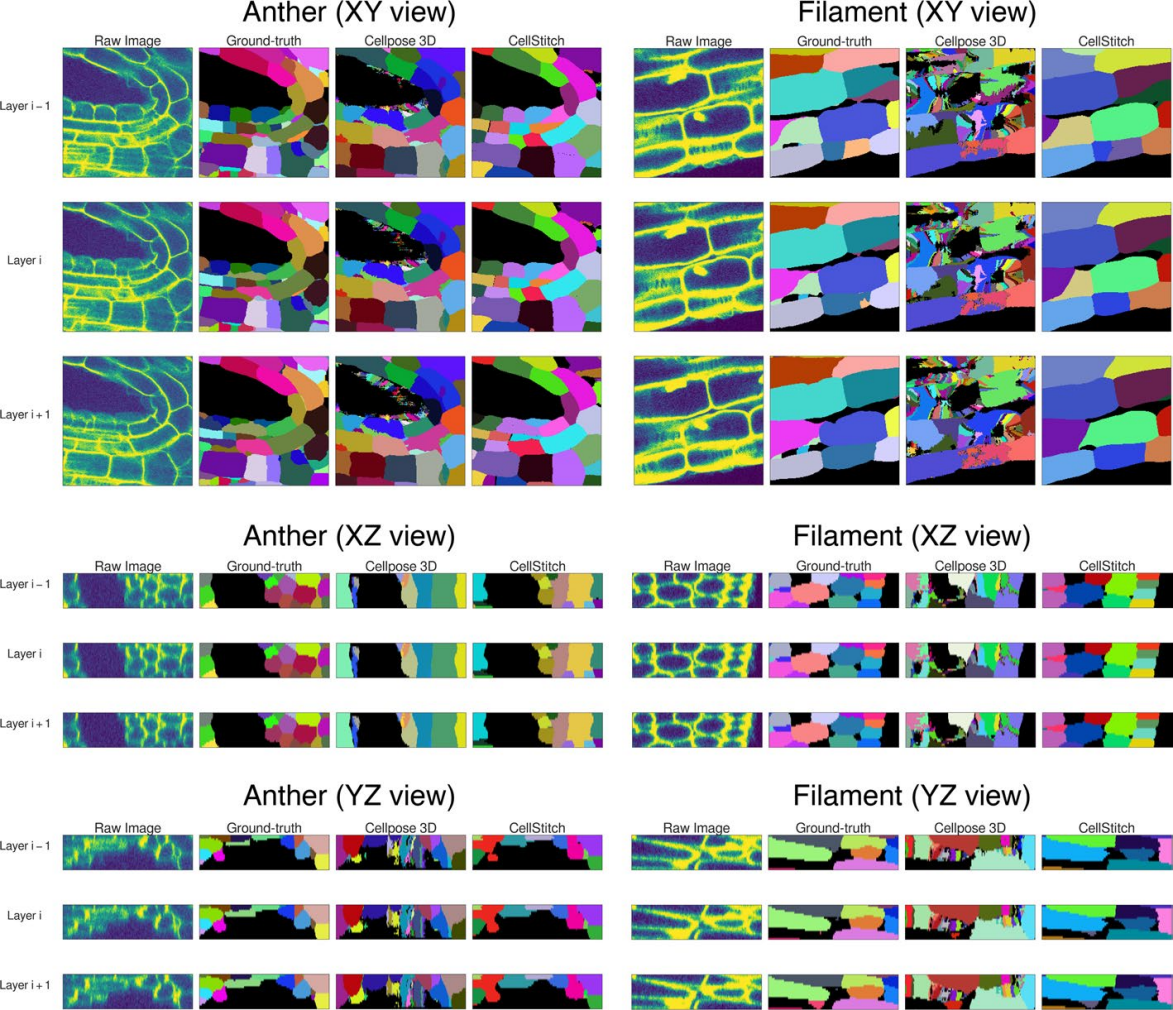
Pipeline results on Arabidopsis 3D Digital Tissue Atlas. Cross-section visualization of raw image, ground-truth annotations, Cellpose3D and CellStitch (left to right) on various tissue regions of Arabidopsis

Finally, to test CellStitch’s performance under the practical setting, where the anisotropy of the datasets might be unknown, we generated six datasets by sampling manually annotated images from the Arabidopsis 3D Digital Tissue Atlas (https://osf.io/fzr56). For each of the six datasets, we trained Cellpose’s 2D segmentation model for 100 epoches with learning rate 0.2 and batch size 8 under a 7-3 train-test split as the backbone to further generate 3D segmentation results for CellStitch and Cellpose3D. We see in Table 4 that CellStitch is consistently the best performing method(s) on all datasets. Furthermore, CellStitch is also able to discover more morphology diversity, whereas Cellpose3D tends to over-segment non-spherical cells (see Fig. 7).

**Table 4:** Performance on Arabidopsis 3D Digital Tissue Atlas

Dataset	Method	Precision	Recall	AP	mAP
Anther	CellStitch$^*$	$\mathbf {0.66 \pm 0.10}$	$\mathbf {0.53 \pm 0.11}$	$\mathbf {0.42 \pm 0.10}$	$\mathbf {0.41 \pm 0.08}$
	Cellpose2D	$0.48 \pm 0.11$	$\mathbf {0.53 \pm 0.10}$	$0.33 \pm 0.08$	$0.34 \pm 0.07$
	Cellpose3D	$0.41 \pm 0.17$	$0.38 \pm 0.13$	$0.24 \pm 0.10$	$0.23 \pm 0.08$
	PlantSeg	$0.31 \pm 0.05$	$0.60 \pm 0.10$	$0.26 \pm 0.04$	$0.26 \pm 0.04$
Filament	CellStitch$^*$	$\mathbf {0.74 \pm 0.14}$	$0.53 \pm 0.12$	$\mathbf {0.46 \pm 0.13}$	$\mathbf {0.46 \pm 0.11}$
	Cellpose2D	$0.51 \pm 0.15$	$\mathbf {0.57 \pm 0.13}$	$0.38 \pm 0.13$	$0.38 \pm 0.12$
	Cellpose3D	$0.03 \pm 0.02$	$0.22 \pm 0.11$	$0.03 \pm 0.02$	$0.03 \pm 0.02$
	PlantSeg	$0.37 \pm 0.08$	$0.31 \pm 0.13$	$0.20 \pm 0.08$	$0.21 \pm 0.06$
Leaf	CellStitch$^*$	$\mathbf {0.83 \pm 0.17}$	$\mathbf {0.75 \pm 0.19}$	$\mathbf {0.68 \pm 0.21}$	$\mathbf {0.66 \pm 0.18}$
	Cellpose2D	$0.65 \pm 0.19$	$\mathbf {0.77 \pm 0.19}$	$0.56 \pm 0.20$	$0.55 \pm 0.18$
	Cellpose3D	$0.07 \pm 0.05$	$0.53 \pm 0.20$	$0.06 \pm 0.04$	$0.06 \pm 0.04$
	PlantSeg	$0.30 \pm 0.08$	$0.71 \pm 0.20$	$0.27 \pm 0.09$	$0.27 \pm 0.08$
Pedicel	CellStitch$^*$	$0.57 \pm 0.23$	$\mathbf {0.44 \pm 0.16}$	$\mathbf {0.35 \pm 0.16}$	$\mathbf {0.36 \pm 0.13}$
	Cellpose2D	$0.36 \pm 0.19$	$\mathbf {0.44 \pm 0.15}$	$0.25 \pm 0.13$	$0.26 \pm 0.11$
	Cellpose3D	$\mathbf {0.61 \pm 0.25}$	$0.30 \pm 0.13$	$0.27 \pm 0.13$	$0.28 \pm 0.09$
	PlantSeg	$0.29 \pm 0.07$	$0.39 \pm 0.13$	$0.20 \pm 0.06$	$0.21 \pm 0.05$
Sepal	CellStitch$^*$	$\mathbf {0.51 \pm 0.12}$	$\mathbf {0.43 \pm 0.16}$	$\mathbf {0.31 \pm 0.12}$	$\mathbf {0.33 \pm 0.09}$
	Cellpose2D	$0.33 \pm 0.11$	$\mathbf {0.44 \pm 0.17}$	$0.23 \pm 0.09$	$0.25 \pm 0.08$
	Cellpose3D	$0.41 \pm 0.23$	$0.34 \pm 0.12$	$0.20 \pm 0.10$	$0.21 \pm 0.09$
	PlantSeg	$0.34 \pm 0.07$	$0.43 \pm 0.14$	$0.23 \pm 0.06$	$0.25 \pm 0.05$
Valve	CellStitch$^*$	$\mathbf {0.71 \pm 0.07}$	$0.47 \pm 0.04$	$\mathbf {0.40 \pm 0.05}$	$\mathbf {0.41 \pm 0.11}$
	Cellpose2D	$0.58 \pm 0.09$	$0.47 \pm 0.04$	$0.35 \pm 0.05$	$0.37 \pm 0.05$
	Cellpose3D$^*$	$0.67 \pm 0.10$	$\mathbf {0.51 \pm 0.05}$	$\mathbf {0.41 \pm 0.06}$	$\mathbf {0.40 \pm 0.05}$
	PlantSeg	$0.53 \pm 0.08$	$0.38 \pm 0.07$	$0.29 \pm 0.06$	$0.32 \pm 0.05$

The best performance (within 0.03) is in bold. The method that achieves the best performance under the majority of the metric is marked with $^*$

## Discussion

We developed CellStitch in order to address the challenge of 3D segmentation of anisotropy images. Existing methods fail to incorporate information from all dimensions (in 2D-based methods), and are not capable of generalizing isotropic frameworks to the anisotropic setting (2.5D-based methods) with the lack of training data (3D-based). Our framework relies on optimal transport to align cell slices from different layers in order to reconstruct 3D segmentation from 2D layers. A limitation of CellStitch and the 2D-based approaches in general is the dependence on the chosen 2D segmentation framework; if the 2D segmentations are unreliable, it is unlikely that CellStitch will produce satisfying results. Due to the rapid development of the deep learning-based 2D segmentation pipeline along with increasing 2D training data, we observe that the 2D segmentation by Cellpose [8] achieves desirable 2D masks on the datasets used in this study—nonetheless, future work will aim to extend CellStitch to further improve existing 2D segmentation methods in order to automate 3D segmentation on more complex tissues.

## Conclusions

Recent advances in imaging and sequencing technologies enable in situ profiling of cell morphology and gene expression in 3D. Deep learning-based 3D segmentation has achieved great success in biomedical imaging [34, 35]. However, various limitations have hindered the wider applications of 3D segmentation to molecular level data, including fluorescence microscopy imaging.

In this paper, we introduce CellStitch, an efficient algorithm to reconstruct 3D cell instances via layer-wise alignment of 2D segmentation results, optionally followed by an instance-wise interpolation modality for users to recover isotropic cell morphologies from highly anisotropic images. CellStitch bridges the gap between the well-studied 2D segmentation problem and the increasing demand for 3D cell segmentation and extends a flexible pipeline to leverage full 3D instance segmentation. Through previous stitching and pipeline benchmarking, CellStitch demonstrated robustness over other 2D, 2.5D, and 3D methods under various anisotropy levels, which is common among shallow-depth 3D microscopic tissue images. Our interpolation results also provide potential insights for in situ experimental designs, where nuclei or cytoplasm signals could be stained in less dense layers than spatial RNA maps, given the cost and technical difficulties to perform multiplexed mRNA, DAPI, and cytoplasm marker imaging.

### Supplementary Information

**Additional file 1**. Supplementary Information.

## Data Availability

A python implementation of this method, CellStitch, is available at https://github.com/imyiningliu/cellstitch. The reproduction of all experiments presented herein can be accessed via https://github.com/imyiningliu/cellstitch/tree/main/notebooks. All the datasets analyzed in this paper are publicly available online. Ovules: https://osf.io/uzq3w/; ATAS: https://www.repository.cam.ac.uk/handle/1810/262530; Arabidopsis 3D Digital Tissue Atlas: https://osf.io/fzr56.

## Abbreviations

2D: Two-dimensional
3D: Three-dimensional
OT: Optimal transport
AP: Average precision
CNNs: Convolutional neural networks
IoU: Intersection-over-Union
ATAS: *Arabidopsis thaliana* apical stem cell
