# Supplementary material for: Cellstitch: 3D cellular anisotropic image segmentation via optimal transport
Source: BMC Bioinformatics. 2023 Dec 15;24:480. doi: 10.1186/s12859-023-05608-2 (PMC10724925; doi:10.1186/s12859-023-05608-2)
Supplement: Supplementary file 1 — Additional file 1. Supplementary Information. [file 12859_2023_5608_MOESM1_ESM.pdf]

Supplementary Materials - CellStitch: 3D Cellular Anisotropic Image Segmentation via Optimal Transport

Yining Liu[1,5], Yinuo Jin[2,5], Elham Azizi[1,2,4,5], Andrew J. Blumberg[1,3,5]

1. Department of Computer Science, Columbia University, New York, USA
2. Department of Biomedical Engineering, Columbia University, New York, USA
3. Department of Mathematics, Columbia University, New York, USA
4. Data Science Institute, Columbia University, New York, USA
5. Irving Institute for Cancer Dynamics, New York, USA

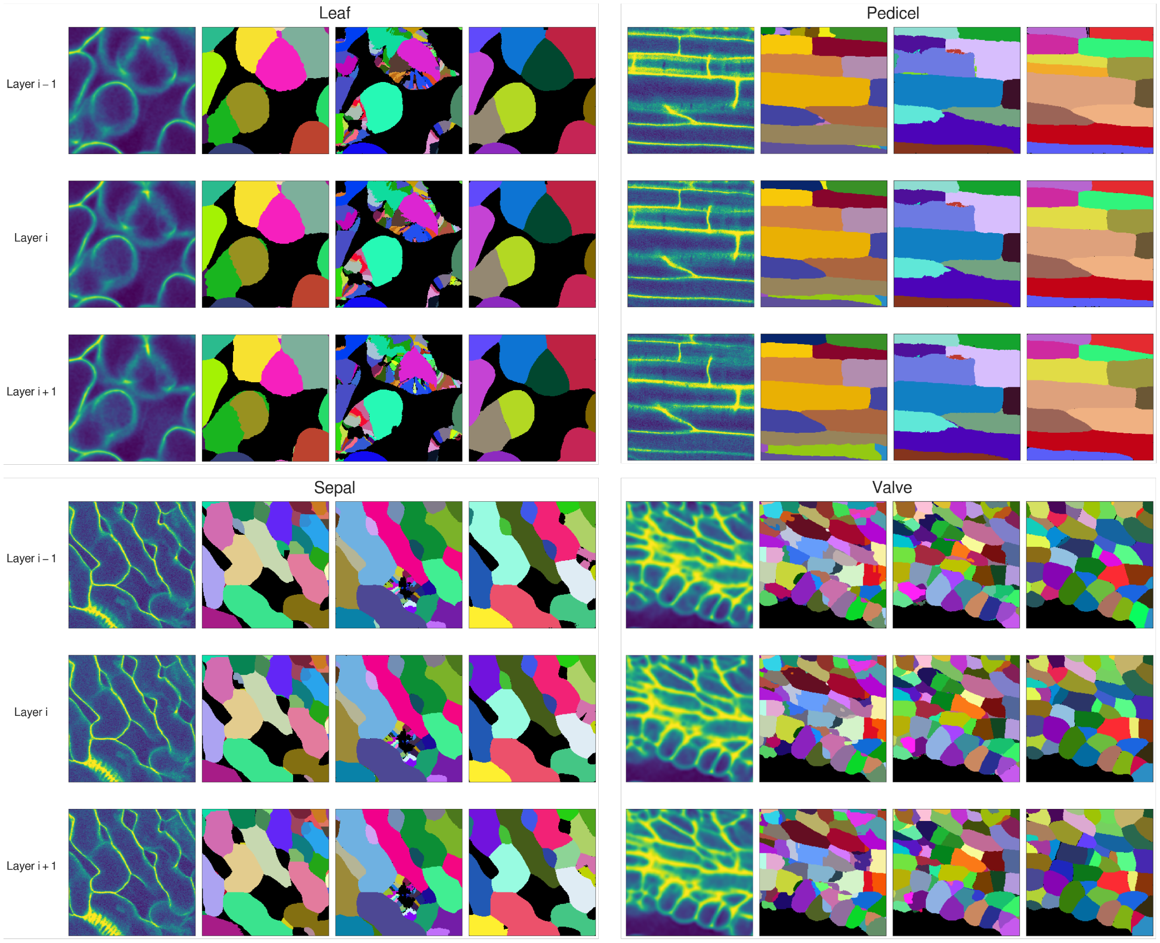

Supplementary Figure 1: Extended visualization of raw image, ground-truth annotations, Cellpose3D & CellStitch (left to right) on various regions of Arabidopsis 3D Digital Tissue Atlas.

Dataset	Method	Precision	Recall	AP	mAP
Ovules	CellStitch*	$\mathbf{0.64 \pm 0.08}$	$\mathbf{0.64 \pm 0.14}$	$\mathbf{0.48 \pm 0.11}$	$\mathbf{0.51 \pm 0.09}$
	3D Watershed	0.07 ± 0.08	0.20 ± 0.18	0.06 ± 0.06	0.06 ± 0.06
ATAS	CellStitch*	$\mathbf{0.79 \pm 0.08}$	$\mathbf{0.77 \pm 0.04}$	$\mathbf{0.64 \pm 0.07}$	$\mathbf{0.70 \pm 0.07}$
	3D Watershed	0.07 ± 0.08	0.07 ± 0.08	0.01 ± 0.01	0.02 ± 0.01
Anther	CellStitch*	$\mathbf{0.66 \pm 0.10}$	$\mathbf{0.53 \pm 0.11}$	$\mathbf{0.42 \pm 0.10}$	$\mathbf{0.41 \pm 0.08}$
	3D Watershed	0.03 ± 0.04	0.03 ± 0.05	0.01 ± 0.02	0.03 ± 0.03
Filament	CellStitch*	$\mathbf{0.74 \pm 0.14}$	$\mathbf{0.53 \pm 0.12}$	$\mathbf{0.46 \pm 0.13}$	$\mathbf{0.46 \pm 0.11}$
	3D Watershed	0.04 ± 0.02	0.15 ± 0.09	0.04 ± 0.02	0.05 ± 0.01
Leaf	CellStitch*	$\mathbf{0.83 \pm 0.17}$	$\mathbf{0.75 \pm 0.19}$	$\mathbf{0.68 \pm 0.21}$	$\mathbf{0.66 \pm 0.18}$
	3D Watershed	0.00 ± 0.00	0.00 ± 0.00	0.00 ± 0.00	0.00 ± 0.00
Pedicel	CellStitch*	$\mathbf{0.57 \pm 0.23}$	$\mathbf{0.44 \pm 0.16}$	$\mathbf{0.35 \pm 0.16}$	$\mathbf{0.36 \pm 0.13}$
	3D Watershed	0.03 ± 0.03	0.04 ± 0.05	0.02 ± 0.02	0.02 ± 0.02
Sepal	CellStitch*	$\mathbf{0.51 \pm 0.12}$	$\mathbf{0.43 \pm 0.16}$	$\mathbf{0.31 \pm 0.12}$	$\mathbf{0.33 \pm 0.09}$
	3D Watershed	0.03 ± 0.05	0.02 ± 0.00	0.01 ± 0.00	0.02 ± 0.00
Valve	CellStitch*	$\mathbf{0.71 \pm 0.07}$	$\mathbf{0.47 \pm 0.04}$	$\mathbf{0.40 \pm 0.05}$	$\mathbf{0.41 \pm 0.11}$
	3D Watershed	0.00 ± 0.00	0.00 ± 0.00	0.00 ± 0.00	0.00 ± 0.00

Supplementary Table 1: Benchmark between CellStitch and 3D Watershed; the **best** performance (within 0.03) is in bold. The method that achieves the best performance under the majority of the metric is marked with *.

Dataset	Method	Precision	Recall	AP	mAP
Ovules	CellStitch*	$\mathbf{0.64 \pm 0.08}$	$\mathbf{0.64 \pm 0.14}$	$\mathbf{0.48 \pm 0.11}$	$\mathbf{0.51 \pm 0.09}$
	Cellpose3D	0.08 ± 0.11	0.56 ± 0.22	0.08 ± 0.11	0.08 ± 0.10
	PlantSeg	0.38 ± 0.05	0.32 ± 0.07	0.21 ± 0.03	0.22 ± 0.03

Supplementary Table 2: Benchmark between competing methods on isotropic upsampled images (Z-axis) and CellStitch on anisotropic images; the **best** performance (within 0.03) is in bold. The method that achieves the best performance under the majority of the metric is marked with *.
